# Analyzing 8-Oxoguanine in Exhaled Breath Condensate: A Novel Within-Subject Laboratory Experimental Study on Waterpipe Smokers

**DOI:** 10.3390/antiox14080929

**Published:** 2025-07-29

**Authors:** Natasha Shaukat, Tarana Ferdous, Simanta Roy, Sharika Ferdous, Sreshtha Chowdhury, Leonardo Maya, Anthony Paul DeCaprio, Wasim Maziak, Taghrid Asfar

**Affiliations:** 1 Department of Epidemiology, Robert Stempel College of Public Health, Florida International University, 11200 SW 8th St, Miami, FL 33199, USA; nshau001@fiu.edu (N.S.); tferd001@fiu.edu (T.F.); siroy@fiu.edu (S.R.); sferd003@fiu.edu (S.F.); srchowdh@fiu.edu (S.C.); wmaziak@fiu.edu (W.M.); 2 Forensic & Analytical Toxicology Facility, Global & Forensic Justice Center, Florida International University, Miami, FL 33199, USA; lmaya@fiu.edu (L.M.); adecapr@fiu.edu (A.P.D.); 3 Department of Public Health Sciences, Miller School of Medicine, University of Miami, Miami, FL 33136, USA; 4 Sylvester Comprehensive Cancer Center, Miller School of Medicine, University of Miami, Miami, FL 33136, USA

**Keywords:** exhaled breath condensate, 8-oxoguanine, oxidative damage, waterpipe

## Abstract

Introduction: This study aimed to analyze exhaled breath condensate (EBC) for 8-oxoguanine (8-oxoGua), an oxidative stress biomarker among waterpipe (WP) smokers. Methods: In a within-subject pre-post exposure design, thirty waterpipe smokers completed two 45 min laboratory sessions. EBC was analyzed for 8-oxoGua before and after WP smoking. Median differences between time points (pre vs. post) were assessed using the Wilcoxon sign rank test, with significance defined as *p* < 0.05. Results: The analysis included 59 WP smoking sessions. Participants had a median age of 24 years (IQR: 21–25), with 62.1% being female. Most had a bachelor’s degree or less (62.1%), and over half were students (55.2%), while 34.5% were employed. The average age for first WP use was 18.6 years, with participants reporting a median of three WP smoking sessions per month. Results indicate a median increase in 8-oxoGua among participants from 5.4 ng/mL (IQR: 8.8) before the smoking session to 7.6 ng/mL after (IQR: 15.7; *p* < 0.001). Conclusions: This study is the first to examine 8-oxoGua in EBC. Findings provide strong evidence of WP smoking’s contribution to oxidative stress in the airways. It justifies the use of EBC to study the exposure to markers of oxidative stress with emerging tobacco use methods such as the waterpipe.

## 1. Introduction

Waterpipe (WP; Hookah) is one of the emerging global tobacco products and in the United States (US), especially among young adults [1,2]. The Centers for Disease Control and Prevention (CDC) reported that in 2018, approximately 7.8% of high school students and 12.3% of young adults aged 19–30 years had used a WP in the previous year [3]. Recent data from the Population Assessment of Tobacco and Health (PATH) study (2013–2021) showed increasing trends in WP smoking among young adults (aged 18–24) compared to other age groups [4]. This trend is alarming, especially given that WP smoking is substantially higher among women and youth compared to older adults [5,6]. Several factors drive the rise in waterpipe smoking: (1) misleading marketing that falsely promotes it as a safer alternative to cigarettes; (2) social acceptance in homes, cafés, and restaurants; (3) its usage normalization through social media; and (4) the lack of waterpipe-specific regulations [1,2,7].

WP smoke contains many toxicants derived mainly from the burning of charcoal and the heating of tobacco [8,9]. These toxicants include carbon monoxide (CO), nicotine, particulate matter, volatile organic chemicals such as formaldehyde and acrolein, arsenic, and heavy metals like lead [8,9,10,11]. When inhaled, these toxicants generate excessive reactive oxygen species (ROS), which are known to cause DNA damage [12,13]. When the level of ROS exceeds the body’s antioxidant defense response, it disrupts the normal redox balance, leading to the generation of oxidative stress [14]. Oxidative stress is one of the potential mechanisms by which smoking predisposes to a range of health problems, such as respiratory conditions (asthma, chronic bronchitis, and cystic fibrosis), cardiovascular diseases, neurological disorders, and cancer [1,8,9,15,16,17].

Previous research has focused on identifying oxidative stress markers in various tobacco-related diseases [8,13,14,17,18]. Among those, 8-oxoguanine (8-oxoGua), an oxidation product of DNA’s guanine base, is a biomarker of ROS-mediated DNA damage [14]. Only a few studies have investigated 8-oxoGua levels in the blood and urine of WP smokers [19,20]. For instance, Jebai et al. found higher urinary 8-oxoGua concentrations after WP smoking [19]. However, due to delayed effects and influences from other sources like environmental factors and nutrition, and complex interactions affecting systemic levels of markers of oxidative stress, it is difficult to determine the precise impact of WP smoking on 8-oxoGua levels. Furthermore, direct airway sampling could clarify this, but traditional methods such as bronchoscopy are often invasive and costly [21]. Therefore, developing non-invasive, site-specific tools to assess early lung changes after WP exposure, especially in young users, presents a critical opportunity.

The main objective of this study is to measure 8-oxoguanine (8-oxoGua), an oxidative stress marker in the exhaled breath condensate (EBC) of young waterpipe smokers using a within-subject pre-post WP smoking session design. EBC is a collection of aerosolized droplets from exhaled air that contains thousands of identified metabolites [21]. It is an easy-to-collect, non-invasive, and affordable biological matrix for sampling airways [22]. Measuring 8-oxoGua in EBC may therefore serve as a non-invasive method to understand the molecular pathological changes occurring in the respiratory tract following WP smoking [21,22]. The analysis of EBC allows us to directly link WP smoking to changes in oxidative stress within the respiratory tract, a method that has not been explored in prior studies.

## 2. Methods

### 2.1. Participants

Thirty current WP smokers (defined as smoking WP at least once a week in the past six months) aged 21–35 years were recruited from Miami, Florida, via flyers, word of mouth, and online advertisements. We excluded participants with self-reported history of chronic health concerns or psychiatric conditions, regular use of prescription medications (other than vitamins or birth control), and current use of ≥ 5 cigarettes or other tobacco/nicotine products in the past month. Women were excluded if they were breastfeeding or tested positive for pregnancy (by urine pregnancy testing) at the screening. Participants were preliminarily screened on the phone for potential eligibility, and the information was later confirmed during an in-person screening session. The Institutional Review Board of Florida International University (FIU) approved this study, and written informed consent was obtained from participants prior to their participation. Participants were compensated for their time, effort, and expenses associated with attending the lab sessions with a $100.

### 2.2. Study Design

All participants completed two WP smoking sessions in the clinical lab at Florida International University (FIU), preceded by ≥12 h tobacco abstinence confirmed by expired Carbon Monoxide (eCO) < 6 ppm. The sessions were separated by at least a 48 h washout period. All participants selected their preferred tobacco flavor from a variety of options offered and smoked the same flavor in both sessions. Before smoking, the WP was prepared by filling the head with 15 g of tobacco product. The head was then covered with perforated foil. Tobacco was heated by two pieces of quick-lighting charcoal lit by study staff and placed on top of the foil. Participants smoked ad libitum for up to 45 min. Participants were seated in a private room with a comfortable reclining chair, given a choice to use any electronic device, and instructed to use WP at their natural pace. The same standardized instructions were given to all participants, and there was limited interaction with participants during the WP smoking session.

### 2.3. EBC Collection

EBC samples were collected 10 to 15 min before and after each WP smoking session. The participants were asked to breathe for 15 min (tidal breathing) through a single-use disposable RTubeTM collector (Respiratory Research, Inc., Charlottesville, VA, USA). After sample collection, a plunger was used to pool the condensed material within a cryogenic vial (about 1–2 mL) and stored at −80 °C within approximately 5 min to minimize degradation and ensure strict sample integrity.

## 3. Measures

### 3.1. Participant Characteristics

Demographic information, including age (years), gender (male/female), race (white/non-white), ethnicity (Hispanic/non-Hispanic), and education status (high school or less/some college or associate degree, bachelor’s degree/more than bachelor degree), employment status (employed, not employed) were collected at baseline.

### 3.2. Waterpipe Pattern Use

WP-related Measures included: age at first WP smoking experience (years); the average number of hookahs (heads/bowls) smoked per month; usual type of WP tobacco smoked (flavored or unflavored); perceived differences in smoke production between flavored and unflavored tobacco (flavored, unflavored, or no difference); average duration of a typical WP smoking session (less than 30 min, 30–60 min, or more than 60 min); shared WP with others (yes/no); and usual smoking place (home, friend’s home, public places such as cafés/restaurants, others).

### 3.3. 8-Oxoguanine Measurements

EBC samples were analyzed by the Forensic and Analytical Toxicology Facility at FIU using Liquid Chromatography-Triple Quadrupole Mass Spectrometry (LC-QqQ-MS) [22]. Before analysis, samples were thawed and vortexed. A 500 µL sample aliquot was then transferred to a plastic Eppendorf tube. A 4 h vacufuge was performed to evaporate all the EBC matrix. The samples were then reconstituted in 75 µL of acetonitrile and spiked with 8-Oxo-2′-deoxyguanosine-[13C,15N2] as an internal standard to facilitate the quantification of 8-oxo-7,8-dihydroguanine.

The limit of detection (LOD) and limit of quantitation (LOQ) for 8-oxo-7,8-dihydroguanine were 5.2 and 15.7 ng/mL, respectively. The LOD and LOQ were calculated using the signal-to-noise ratio of 3:1 for LOD and 10:1 for LOQ. The calculations were based on three replicates per calibration point. The 8-oxoguanine measurement was validated using a fit-for-purpose approach. Validation experiments were conducted in both acetonitrile and EBC to assess method performance. Carryover, matrix effects, and interference studies were performed to ensure specificity and reliability in batch analysis. Calibration curves were generated in triplicate to determine the LOD and LOQ, and to assess linearity. All calibration curves demonstrated strong linearity, with R^2^ values consistently exceeding 0.99. No matrix effects, carryover, or interference between the analyte and other compounds were observed [23].

### 3.4. Data Analysis

Baseline characteristics of the study sample were summarized using means and standard deviations (SDs) for continuous variables, along with proportions for categorical variables. Pre- and post-concentrations of 8-oxoGua for each session were calculated and presented with medians and interquartile ranges (IQRs). Planned comparisons were conducted with the Wilcoxon signed-rank test to examine median differences between time points (pre vs. post). Data were analyzed using SPSS version 25, and a *p*-value greater than 0.05 was considered statistically significant.

## 4. Results

The analysis included 59 WP smoking sessions. Table 1 shows the sociodemographic and WP use characteristics of the study participants (n = 30). The median age was 24 years (IQR: 21–25), and the majority were female (62.1%). Most participants had a bachelor’s degree or less (62.1%), and over half were students (55.2%), with 34.5% employed.

The mean age at first WP use was 18.6 years (SD = 2.4). Participants reported a median of 3 WP smoking sessions per month (IQR: 1–5). Regarding typical session duration, 62.1% reported smoking for 30–60 min, 20.1% for less than 30 min, and 17.2% for more than 60 min.

**Figure 1:**
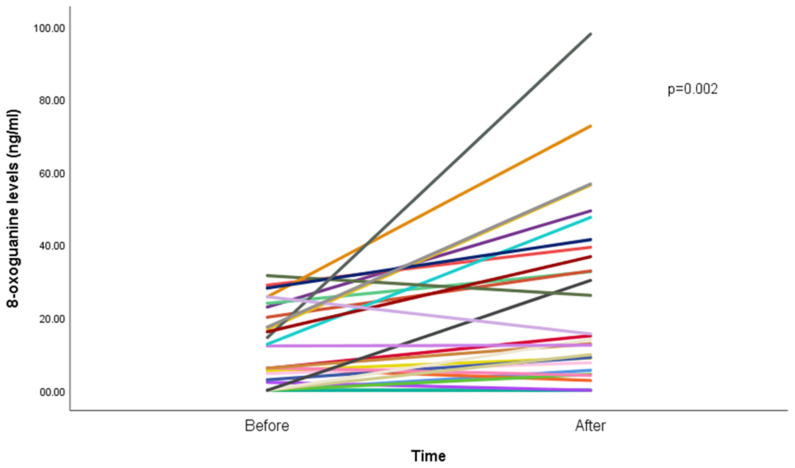
8-oxoguanine concentration (ng/mL) before and after waterpipe smoking (n = 29). Each line represents a participant; overlapping lines reflect similar values. One participant was excluded due to missing post-session data.

Results show an increase in median 8-oxoGua among the participants, from 5.4 ng/mL (IQR: 8.8) before the WP smoking session to 7.6 ng/mL (IQR: 15.7; *p* < 0.001) after the WP smoking session (Figure 1).

## 5. Discussion

This study is the first to assess 8-oxoGua levels in EBC before and after a waterpipe (WP) smoking session in young adults. We observed a statistically significant increase in EBC 8-oxoGua concentrations post-smoking, indicating acute oxidative DNA damage in the lower respiratory tract [21]. These within-subject findings provide strong evidence of WP’s immediate impact on the respiratory airways, using a non-invasive biomarker that reflects local airway-level oxidative stress. This biomarker-driven approach significantly enhances early detection of oxidative stress in the lungs and is essential for advancing clinical trials and enforcing tobacco regulation. The concerning evidence of DNA damage in the airways underscores the urgent need for targeted WP-specific policies and comprehensive educational campaigns.

Direct assessment of airway inflammation is performed using invasive techniques such as induced sputum, bronchoalveolar lavage fluid collection, or bronchial biopsy. In contrast, exhaled breath condensate (EBC), obtained by cooling exhaled air during normal breathing, is a simple, noninvasive, and safe [24]. While saliva is a convenient and fully non-invasive matrix for measuring oxidative stress markers, it predominantly reflects oral cavity and systemic sources and can be influenced by diet, oral hygiene, and local inflammatory conditions [21,25]. In contrast, EBC samples the airway lining fluid and provides a more specific assessment of lower respiratory tract oxidative stress [24], making it particularly suitable for evaluating inhalation-related exposures such as waterpipe smoking. Several studies have shown an increase in 8-oxoGua in lung tissue, plasma, and urine of cigarette and waterpipe smokers in comparison to nonsmokers [19]. For example, a previous study demonstrated that the urinary concentration of 8-oxoGua increased significantly after smoking flavored waterpipe tobacco (from 2.96 ± 0.84 to 3.45 ± 0.76 ng/mg creatinine, *p* = 0.003) [19]. Our results are consistent with these findings, indicating a direct oxidative damage potential of WP smoking on the airway of smokers. Our new method of measuring 8-oxoGua using exhaled breath condensate (EBC) offers significant advantages over traditional urinary or plasma samples. Notably, it has the potential to eliminate systemic exposures and interactions that can interfere with the measurement of 8-oxoGua in those samples. This includes influences from chemical agents, ionizing and UV radiation, as well as environmental pollutants.

The study has several limitations, including a relatively small sample size of young adults. Despite the small sample size, we had sufficient statistical power to identify a significant difference in 8-oxoGua levels, a biomarker of oxidative stress, before and after waterpipe (WP) smoking. Additionally, the laboratory setting differs from the typical environment in which people smoke waterpipes, which may influence smoking patterns and exposure levels. To mitigate this issue, we made efforts to create a comfortable atmosphere by allowing participants to sit in reclining chairs while smoking a WP and watching movies of their choice. Another limitation is that we did not include repeated measurements of 8-oxoGua levels to assess intra-individual reproducibility, as this was not an objective of the present study. Furthermore, our study focused solely on the acute effects of waterpipe smoking; future research should explore the long-term impact of repeated WP smoking on oxidative stress using longitudinal designs. Finally, since we did not include nonsmokers in our study, we are unable to compare our results with those of nonsmokers or never-smokers. Incorporating non-smoker controls would provide a more comprehensive comparison across different demographic groups. Additionally, evaluating a wider range of oxidative stress biomarkers in EBC, such as 8-isoprostane or malondialdehyde, could provide deeper insights into the mechanisms of harm related to waterpipe use. Such evidence would help inform targeted risk reduction strategies and support the development of standardized protocols for EBC biomarker analysis in tobacco research.

## 6. Conclusions

In summary, while evidence of the harms of WP smoking is growing, previous studies have mainly focused on investigating systemic biomarkers, which may overlook localized airway damage. This study demonstrates that WP smoking acutely elevates 8-oxoGua levels in airway lining fluid, indicating localized oxidative DNA damage and highlighting EBC as a valuable, non-invasive method for monitoring respiratory oxidative stress. The results deliver robust and compelling evidence of WP smoking’s contribution to oxidative stress in the airways of smokers. Given the alarming rise in WP use among youth and widespread misconceptions regarding its safety, immediate action is imperative to protect public health.

## Figures and Tables

**Table 1:** Sociodemographic and waterpipe use-related characteristics of the participants (n = 29) *.

Variables	N (%)
Sex	
Females	18 (62.1)
Males	11(37.9)
Age in years, Median (IQR)	24 (21.25)
Education	
Bachelor’s or less	18 (62.1)
Master’s and above	11(37.9)
Employment Level	
Employed	10 (34.5)
Not employed	3 (10.3)
Student	16 (55.2)
Age at WP first use, Mean (SD)	18.6 (2.4)
WP month average, Median (IQR)	3 (1.5)
Average time spent on WP smoking session	
Less than 30 min	6 (20.1)
30–60 min	18 (62.1)
More than 60 min	5 (17.2)

* One participant had missing data.

## Data Availability

The data presented in this study are available upon reasonable request from the corresponding author. The data are not publicly available due to privacy/ethical restrictions.
